# One-step synthesis of highly efficient three-dimensional Cd_1-*x*_Zn_*x*_S photocatalysts for visible light photocatalytic water splitting

**DOI:** 10.1186/1556-276X-8-334

**Published:** 2013-07-24

**Authors:** Zuzhou Xiong, Maojun Zheng, Changqing Zhu, Bin Zhang, Li Ma, Wenzhong Shen

**Affiliations:** 1 Key Laboratory of Artificial Structures and Quantum Control (Ministry of Education), Department of Physics and Astronomy, Shanghai Jiao Tong University, Shanghai 200240, People's Republic of China; 2 School of Chemistry and Chemical Technology, Shanghai Jiao Tong University, Shanghai 200240, People's Republic of China

**Keywords:** Visible light photocatalytic, Water splitting, Cd_1−*x*_Zn_*x*_S, Solvothermal pathway, Solid solutions

## Abstract

Visible light accounts for about 43% of the solar spectrum, and developing highly efficient visible-light-driven photocatalyst is of special significance. In this work, highly efficient three-dimensional (3D) Cd_1−*x*_Zn_*x*_S photocatalysts for hydrogen generation under the irradiation of visible light were synthesized via one-step solvothermal pathway. Scanning electron microscope, X-ray diffractometer, Raman spectrometer, and X-ray photoelectron spectrometer were utilized to characterize the morphology, crystal structure, vibrational states, and surface composition of the obtained 3D Cd_1−*x*_Zn_*x*_S. UV-Vis spectra indicated that the as-synthesized Cd_1−*x*_Zn_*x*_S had appropriate bandgap and position of the conduction band that is beneficial for visible light absorption and photo-generated electron-hole pair separation. Moreover, the 3D structure offers a larger surface area thus supplying more surface reaction sites and better charge transport environment, and therefore, the efficiency of water splitting was improved further.

## Background

The efficient conversion of solar energy into fuel via photochemical reactions is of great importance for the next-generation energy source for its cleanable, renewable, and abundant properties [1,2]. Solar-hydrogen, the conversion of solar energy into hydrogen as chemical energy carrier, has been regarded as one of the most desirable ways in considering energy consumption, resource sustainability, and environmental issues [3,4].

Since the pioneering work of Fujishima and Honda in 1972 [5], tremendous research on semiconductor-based photocatalysis and photoelectrolysis has yielded a better understanding of the mechanisms involved in photocatalytic and photoelectrochemical water splitting [6-9]. However, most of semiconductor photocatalysts can only absorb ultraviolet light due to their wide gap. As it is well known, ultraviolet light occupies only 3% ~ 5% of the solar spectrum; so, the energy conversion efficiency is usually very low [10-12]. Thus, exploiting of highly active visible-light-responsive photocatalysts to make the best use of solar energy in visible light region, which accounts for about 43% of the solar spectrum, is particularly important [13,14]. In the past, developing and understanding of semicondutor electrodes or photocatalysts for photoelectrochemical or photocatalytic water splitting were mainly performed on simple binary systems (e.g., binary oxides [15,16] and chalcogenides [17,18]) and their composite structure [19]. Recently, the ternary system as potentially excellent photoelectrode or photocatalyst material has attracted more and more attention [20-22] because ternary system can offer more possibilities for bandgap and band position tuning.

Cadmium sulfide is an important visible-light response photocatalytic material, in which sulfide ions serve as electron donors. However, the sulfide ion is readily oxidized to sulfate by the photo-generated holes, with Cd^2+^ ions escaping into the solution. A feasible way for enhancing the photocatalytic activity and stability of cadmium sulfide is to develop CdS-based composite materials. Zinc sulfide has the similar crystal structure as cadmium sulfide. It is a good host material for the development of a visible-light-driven photocatalyst without adding noble metals by forming Cd_1−*x*_Zn_*x*_S solid solutions with a narrow bandgap semiconductor, CdS [22,23]. The bandgap of the solid solutions formed between ZnS and CdS can be regulated by changing the compositions and therefore the photocatalytic properties can be varied [24,25].

In this article, we reported a highly efficient three-dimensional (3D) visible-light-active Cd_1−*x*_Zn_*x*_S photocatalysts synthesized via one-step solvothermal pathway. The obtained photocatalysts had good crystallinity and ordered structure and showed excellent photocatalytic activity under the irradiation of visible light.

## Methods

### Synthesis of photocatalyst

Three-dimensional Cd_1−*x*_Zn_*x*_S nanowires were synthesized in a Teflon-lined stainless steel cylindrical closed chamber with a 100-mL capacity. All the chemicals were of analytical grade. Ethylenediamine (en; 60 ml) and H_2_O (20 ml) were used as solvent. Thiourea [NH_2_CSNH_2_] (15 mmol) was added into the solvent as sulfur source, then 5-mmol mixture of cadmium acetate [(CH_3_COO)_2_Cd·2H_2_O] and zinc acetate [(CH_3_COO)_2_Zn·2H_2_O] was added into the mixed solution. After stirring for a few minutes, the closed chamber was placed inside a preheated oven at 160°C for 10 h and then cooled to room temperature. The obtained precipitates were filtered off and washed several times with water and ethanol, respectively. The final products were dried in vacuum at 45°C for a few hours.

### Characterization

The morphology of the as-synthesized powder products were observed by field-emission scanning electron microscopy (Philips Sirion 200, Philips, Netherlands). The crystallographic structure was determined by X-ray diffraction (XRD, D8 DISCOVER X-ray diffractometer, Bruker, Karlsruhe, Germany) with Cu Kα radiation (1.54 Å). Surface composition of the sample was analyzed by X-ray photoelectron spectroscopy (XPS, AXIS ULTRA DLD, Kratos, Japan). The Raman spectrum was measured by the Jobin Yvon LabRam HR 800 UV system (Horiba, Kyoto, Japan) at room temperature. A laser wavelength of 514.5 nm was used as the excitation sources. Reflectance spectra of the obtained were collected using a UV/vis spectrometer (Lambda 20, Perkin Elmer, Inc., USA).

### Photocatalytic hydrogen evolution

The photocatalytic performance of the synthesized 3D Cd_1−*x*_Zn_*x*_S photocatalysts were investigated in a gas-closed circulation system (Labsolar-III, Beijing Perfactlight Technology Co. Ltd., Beijing, China) with a top-window Pyrex cell. A 300-W Xe lamp (SOLAREDGE700, Beijing Perfactlight Technology Co. Ltd., Beijing, China) was used as the light source, and UV light was removed by a cut-off filter (*λ* > 420 nm). Luminous power of the light source is about 40 W. The amount of H_2_ evolved was analyzed by an online gas chromatography (GC7900, Techcomp Ltd., Beijing, China) equipped with a thermal conductivity detector, MS-5A column, and N_2_ was used as carrier. In all experiments, 100 mL deionized water containing the mixed sacrificial agent which composed of 0.25 M Na_2_SO_3_ and 0.35 M Na_2_S were added into the reaction cell. Then, these photocatalysts were directly placed into the electrolyte solution. The whole system was vacuumized with a vacuum pump before reaction to remove the dissolved air. The temperature for all photocatalytic reactions was kept at about 20°C.

## Results and discussions

**Figure 1:**
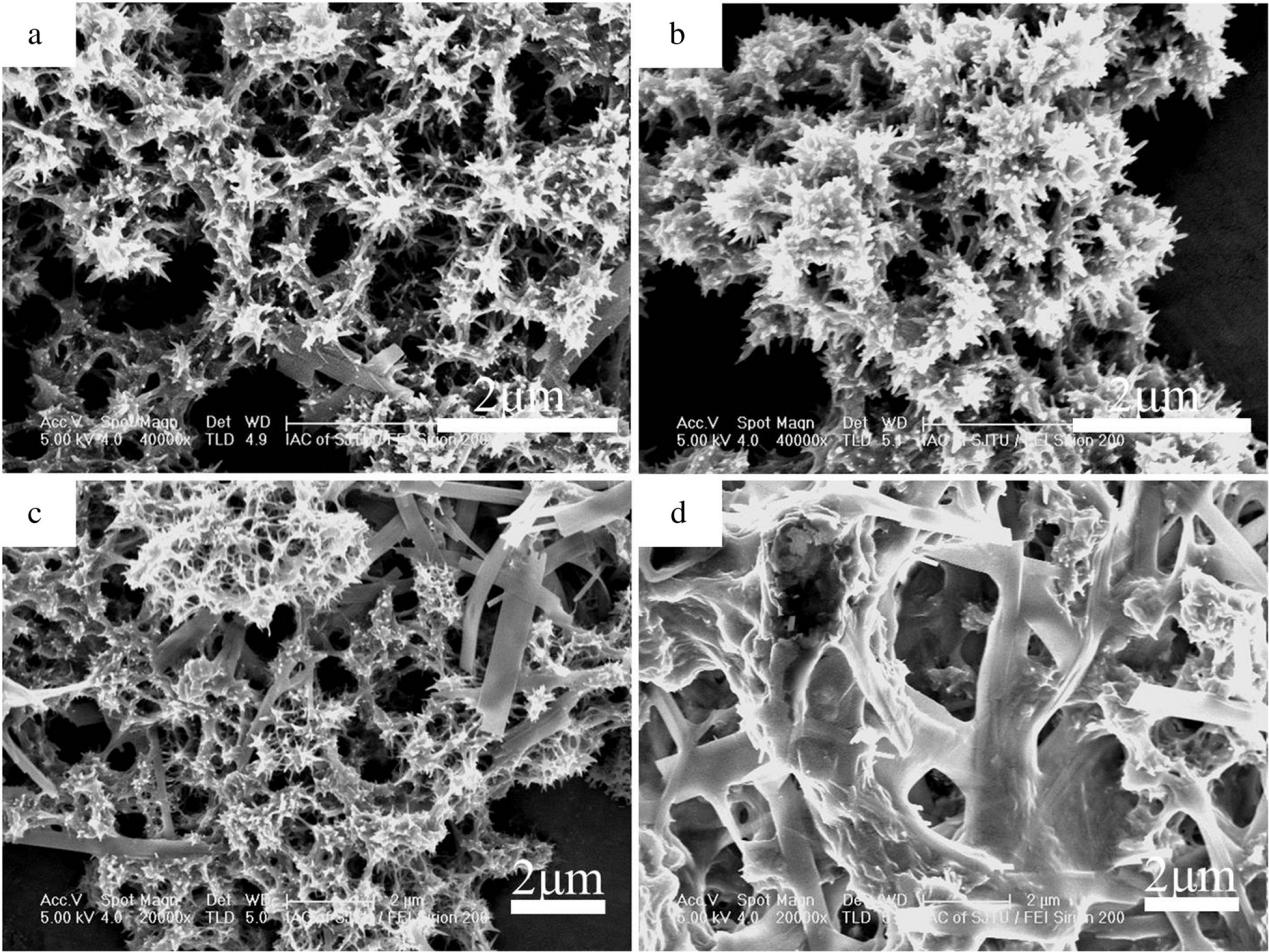
**Typical SEM images of the obtained Cd**_**1−*x***_**Zn**_***x***_**S photocatalysts. (a)** Cd_0.98_S, **(b)** Cd_0.9_Zn_0.1_S, **(c)** Cd_0.72_Zn_0.26_S, and **(d)** Cd_0.24_Zn_0.75_S.

**Figure 2:**
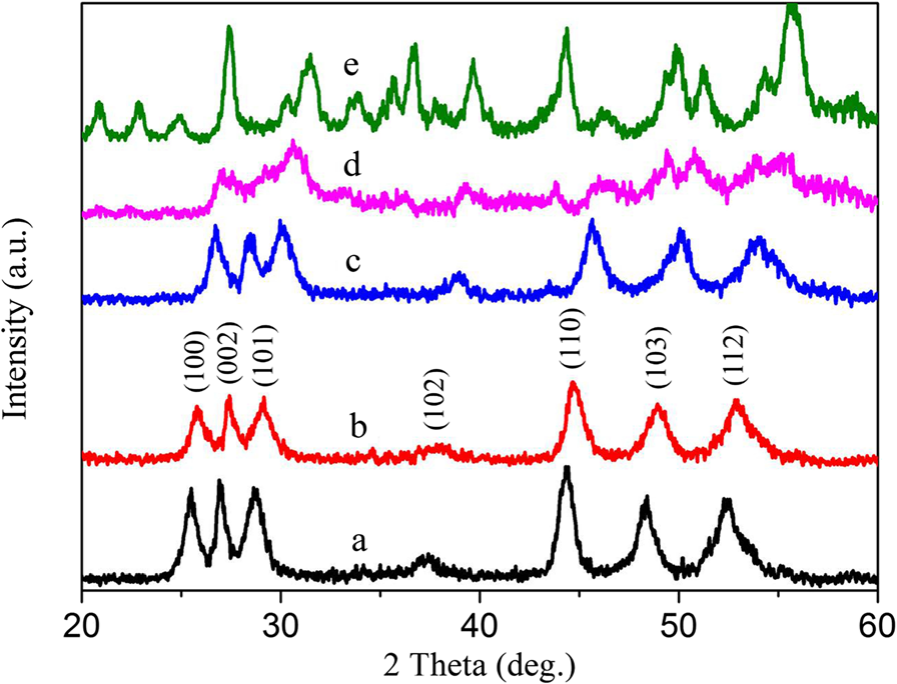
**XRD patterns of the as-prepared Cd**_**1−*x***_**Zn**_***x***_**S photocatalysts with different *x *values. (curve a**) Cd_0.98_S, **(curve b**) Cd_0.9_Zn_0.1_S, **(curve c**) Cd_0.72_Zn_0.26_S, **(curve d**) Cd_0.24_Zn_0.75_S, and **(curve e**) Zn_0.96_S.

The surface morphologies of the obtained Cd_1−*x*_Zn_*x*_S are shown in Figure 1. Figure 1a is the scanning electron microscopy (SEM) image of CdS; it presents porous flower-like 3D structure clearly, shorter nanowires appear at the periphery. As the value of *x* increases, nanosheet emerges gradually, that is, the secondary structure builds up slowly. Figure 2 shows the XRD patterns of the as-prepared photocatalysts. CdS exhibits a Greenockite structure, while ZnS presents a Wurtzite polycrystalline structure, respectively. The diffraction peaks of the photocatalysts shift to a higher angle side as the value of *x* increases. The successive shift of the XRD patterns means that the crystals obtained are Cd_1−*x*_Zn_*x*_S solid solution, not a simple mixture of ZnS and CdS [26].

**Figure 3:**
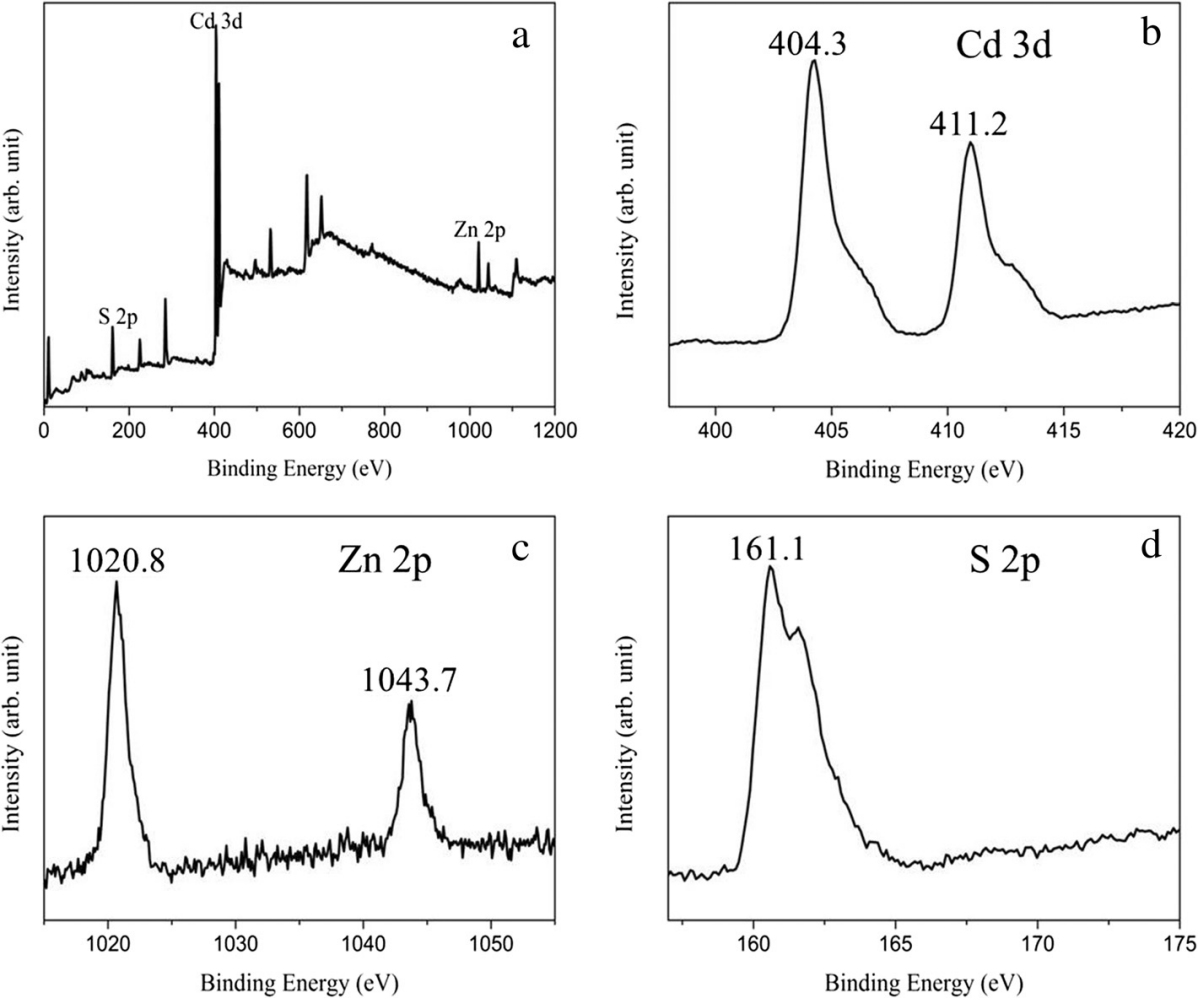
**Representative XPS spectra of typical sample Cd**_**0.72**_**Zn**_**0.26**_**S. (a)** survey spectrum, **(b)** Cd 3d XPS spectrum, **(c)** Zn 2p XPS spectrum, and **(d)** S 2p XPS spectrum.

The surface information is collected by XPS of the sample Cd_0.72_Zn_0.26_S (Figure 3). The survey scan spectrum (Figure 3a) indicates the existence of Cd, Zn, and S in the Cd_0.72_Zn_0.26_S sample. The two sharp peaks (Figure 3b) located at 404.3 and 411.2 eV are corresponding to the Cd 3d_5/2_ and Cd 3d_3/2_ level, respectively. The peaks of 1,020.8 and 1,043.7 eV can be assigned to the Zn 2p_3/2_ and 2p_1/2_ levels, respectively (Figure 3c). The single S 2p peak at 161.1 eV (Figure 3d) demonstrates that sulfur exists as a sulfur ion.

**Figure 4:**
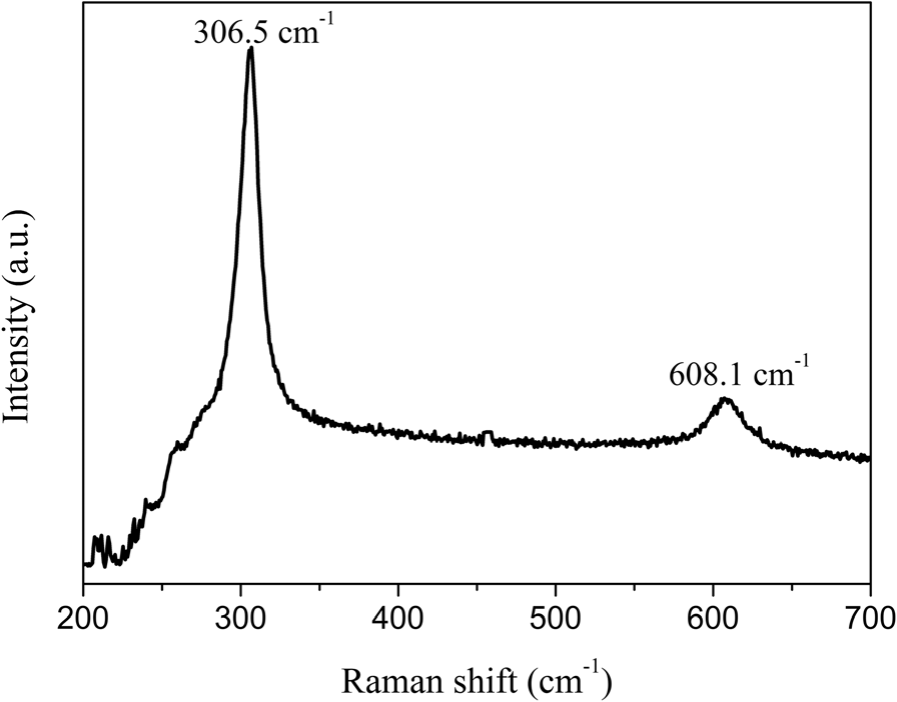
**Raman spectrum of the typical sample Cd**_**0**.**72**_**Zn**_**0**.**26**_**S.**

Raman scattering is a nondestructive technique for structural study of the material and a powerful probe to obtain the vibrational states of a solid. It is an inelastic process in which incoming photons exchange energy with the crystal vibrational mode. Figure 4 reveals the Raman spectrum of the as-obtained Cd_0.72_Zn_0.26_S sample. Bulk CdS has two characteristics of longitudinal-optical (LO) phonon peaks: (1) 1-LO (first harmonic (at 300/cm)) and (2) 2-LO (second harmonic (at 600/cm)) vibrations [27]. The two phonon peaks are also observed in the as-obtained Cd_0.72_Zn_0.26_S; they are located at 306.5 and 608.1/cm, respectively, and shift toward the higher energy side compared with that of the pure CdS. This can be ascribed to the Cd → Zn substitution in the obtained nanophotocatalysts. In addition, from Figure 4, the Raman intensities of 1-LO and 2-LO are both relatively strong and narrow, which implies its good crystallinity and ordered structure [28].

**Figure 5:**
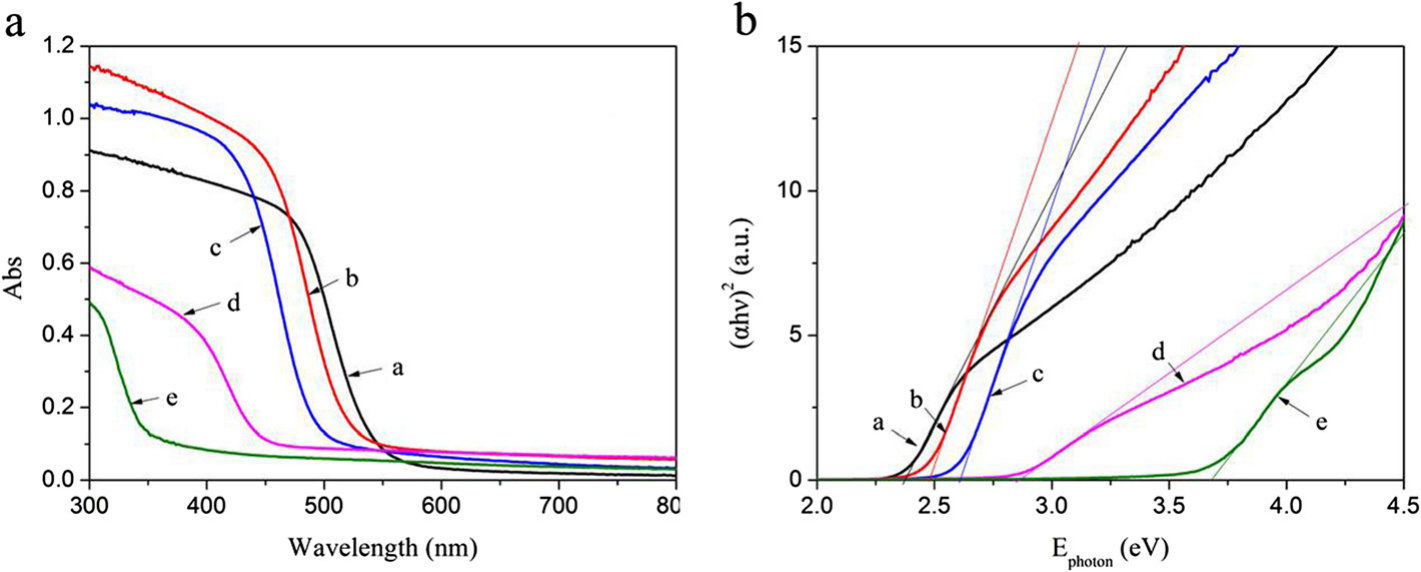
**UV**-**vis absorption spectra (a) and bandgap evaluation (b) from the plots of** (***αE***_**photon**_)^**2 **^**vs**. ***E***_**photon.**_ (curve a) Cd_0.98_S, (curve b) Cd_0.9_Zn_0.1_S, (curve c) Cd_0.72_Zn_0.26_S, (curve d) Cd_0.24_Zn_0.75_S, and (curve e) Zn_0.96_S, respectively.

Curves a, b, c, d, and e of Figure 5 show the UV-vis absorption spectra of the as-prepared Cd_0.98_S, Cd_0.9_Zn_0.1_S, Cd_0.72_Zn_0.26_S, Cd_0.24_Zn_0.75_S, and Zn_0.96_S, respectively. The absorption edge of Cd_1−*x*_Zn_*x*_S solid solutions are red-shifted relative to ZnS (Figure 5a), which can be attributed to the incorporation of Zn into the lattice of CdS or entered its interstitial sites (the radii of Zn^2+^ ion (0.74 Å) is smaller than that of Cd^2+^ (0.97 Å)). The bandgap of Cd_1−*x*_Zn_*x*_S can be acquired from plots of (*αE*_photon_)^2^ versus the energy (*E*_photon_) of absorbed light (*α* and *E*_photon_ are the absorption coefficient and the discrete photon energy, respectively). The extrapolated value (a straight line to the *x*-axis) of *E*_photon_ at *α* = 0 gives absorption edge energies corresponding to *E*_g_. From Figure 5b, the bandgap of the synthesized Cd_1−*x*_Zn_*x*_S are 2.37 eV (curve a), 2.48 eV (curve b), 2.60 eV (curve c), 2.86 eV (curve d), and 3.67 eV (curve e), respectively. The bandgaps of Cd_1−*x*_Zn_*x*_S are beneficial to absorbing solar light to drive the water splitting reaction.

**Figure 6:**
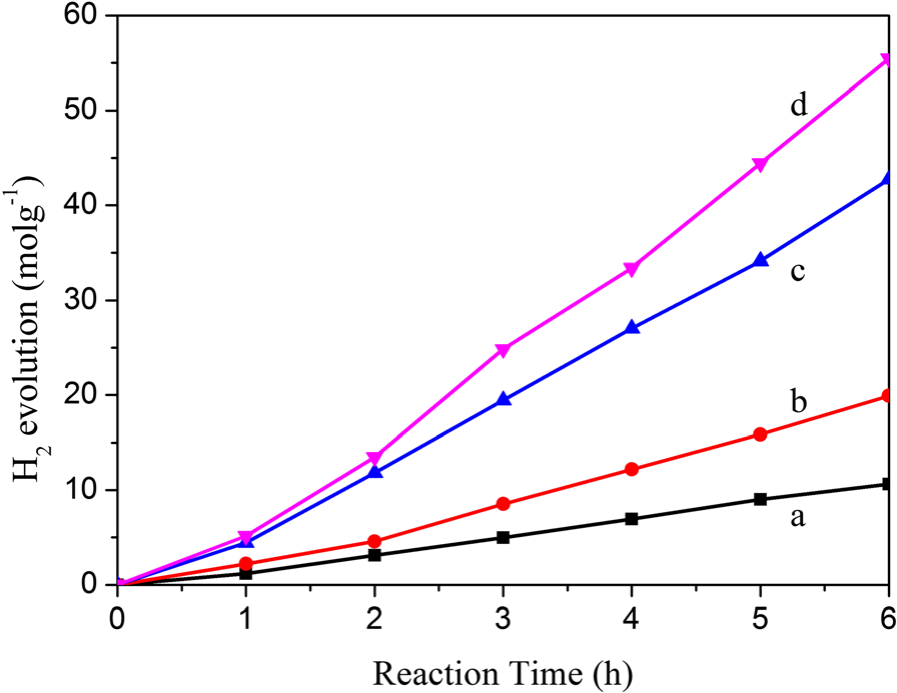
**Photocatalytic H**_**2 **_**evolution of the obtained Cd**_**1**−***x***_**Zn**_***x***_**S photocatalysts.** (curve **a**) Cd_0.98_S, (curve **b**) Cd_0.9_Zn_0.1_S, (curve **c**) Cd_0.72_Zn_0.26_S, and (curve **d**) Cd_0.24_Zn_0.75_S.

The photocatalytic hydrogen evolution of the obtained 3D Cd_1−*x*_Zn_x_S photocatalysts under the irradiation of visible light is given in Figure 6. All of the Cd_1−*x*_Zn_*x*_S photocatalysts show much higher photocatalytic H_2_ evolution capacity than that of the sole CdS at visible light irradiation (*λ* > 420 nm). In addition, the photocatalytic activity of the Cd_1−*x*_Zn_*x*_S solid solutions is strongly dependent on the composition of the solid solutions. It is improved obviously with the increase of Zn content (*x* value). When the *x* value increases to 0.75, the 3D solid solutions photocatalyst has the highest photocatalytic activity. This is because ZnS has a high energy conversion efficiency, it is a good host material for the development of a visible-light-driven photocatalyst by forming solid solutions with a narrow bandgap semiconductor, CdS. The more negative reduction potential of the conduction band of solid solutions would allow for more efficient hydrogen generation than CdS. In addition, the large bandgap and wide valence bandwidth benefit the separation of the photo-generated electrons and holes, and the photocorrosion of the photocatalysts can be reduced effectively. The highest activity probably means that Cd_0.24_Zn_0.75_S has an optimum bandgap and a moderate position of the conduction band, beneficial for visible light absorption and photo-generated electron-hole pair separation. Moreover, the 3D structure offers a larger surface area, thus supplying more surface reaction sites and better charge transport environment. Therefore, the efficiency of water splitting is improved further. It is worth noting that no H_2_ was detected for ZnS photocatalyst because its bandgap is too large to absorb the visible light.

## Conclusions

We reported highly efficient three-dimensional Cd_1−*x*_Zn_*x*_S photocatalysts synthesized via one-step solvothermal pathway for photocatalytic H_2_ evolution under the irradiation of visible light. The Raman spectrum implied the obtained Cd_1−*x*_Zn_*x*_S had good crystallinity and ordered structure. The XPS demonstrated that sulfur existed as a sulfur ion, while Cd and Zn are in 3d and 2p state, respectively. The bandgap of the synthesized Cd_1−*x*_Zn_*x*_S varied from 2.37 to 2.86 eV, which were suitable for the absorption of visible light. The photocatalytic activity of the obtained Cd_1−*x*_Zn_*x*_S photocatalysts were improved markedly compared with that of the sole CdS. This can be attributed to their appropriate bandgap and position of the conduction band that is beneficial for visible light absorption and photo-generated electron-hole pair separation, as well as 3D structure that offered a larger surface area, thus supplying more surface reaction sites and better charge transport environment.

## Abbreviations

3D: Three-dimensional; XRD: X-ray diffraction; XPS: X-ray photoelectron spectrometer.

## Competing interests

The authors declare that they have no competing interests.

## Authors' contributions

ZZX participated in the design of the study, carried out the experiments, and performed the statistical analysis, as well as drafted the manuscript. MJZ participated in the design of the study, provided the theoretical and experimental guidance, performed the statistical analysis, and revised the manuscript. CQZ and BZ helped in the experiments and data analysis. LM participated in the design of the experimental section and offered help in the experiments. WZS gave his help in using the experimental apparatus. All authors read and approved the final manuscript.
